# Genetically encoded X-ray cellular imaging for nanoscale protein localization

**DOI:** 10.1093/nsr/nwaa055

**Published:** 2020-04-02

**Authors:** Huating Kong, Jichao Zhang, Jiang Li, Jian Wang, Hyun-Joon Shin, Renzhong Tai, Qinglong Yan, Kai Xia, Jun Hu, Lihua Wang, Ying Zhu, Chunhai Fan

**Affiliations:** Bioimaging Center, Shanghai Synchrotron Radiation Facility, Zhangjiang Laboratory, Shanghai Advanced Research Institute, Chinese Academy of Sciences, Shanghai 201210, China; Division of Physical Biology, CAS Key Laboratory of Interfacial Physics and Technology, Shanghai Institute of Applied Physics, Chinese Academy of Sciences, Shanghai 201800, China; Bioimaging Center, Shanghai Synchrotron Radiation Facility, Zhangjiang Laboratory, Shanghai Advanced Research Institute, Chinese Academy of Sciences, Shanghai 201210, China; Division of Physical Biology, CAS Key Laboratory of Interfacial Physics and Technology, Shanghai Institute of Applied Physics, Chinese Academy of Sciences, Shanghai 201800, China; Bioimaging Center, Shanghai Synchrotron Radiation Facility, Zhangjiang Laboratory, Shanghai Advanced Research Institute, Chinese Academy of Sciences, Shanghai 201210, China; Division of Physical Biology, CAS Key Laboratory of Interfacial Physics and Technology, Shanghai Institute of Applied Physics, Chinese Academy of Sciences, Shanghai 201800, China; Canadian Light Source Inc., University of Saskatchewan, Saskatoon, SK S7N 2V3, Canada; Pohang Accelerator Laboratory, POSTECH, Pohang 37673, Korea; Bioimaging Center, Shanghai Synchrotron Radiation Facility, Zhangjiang Laboratory, Shanghai Advanced Research Institute, Chinese Academy of Sciences, Shanghai 201210, China; Division of Physical Biology, CAS Key Laboratory of Interfacial Physics and Technology, Shanghai Institute of Applied Physics, Chinese Academy of Sciences, Shanghai 201800, China; Division of Physical Biology, CAS Key Laboratory of Interfacial Physics and Technology, Shanghai Institute of Applied Physics, Chinese Academy of Sciences, Shanghai 201800, China; Bioimaging Center, Shanghai Synchrotron Radiation Facility, Zhangjiang Laboratory, Shanghai Advanced Research Institute, Chinese Academy of Sciences, Shanghai 201210, China; Division of Physical Biology, CAS Key Laboratory of Interfacial Physics and Technology, Shanghai Institute of Applied Physics, Chinese Academy of Sciences, Shanghai 201800, China; Bioimaging Center, Shanghai Synchrotron Radiation Facility, Zhangjiang Laboratory, Shanghai Advanced Research Institute, Chinese Academy of Sciences, Shanghai 201210, China; Division of Physical Biology, CAS Key Laboratory of Interfacial Physics and Technology, Shanghai Institute of Applied Physics, Chinese Academy of Sciences, Shanghai 201800, China; Bioimaging Center, Shanghai Synchrotron Radiation Facility, Zhangjiang Laboratory, Shanghai Advanced Research Institute, Chinese Academy of Sciences, Shanghai 201210, China; Division of Physical Biology, CAS Key Laboratory of Interfacial Physics and Technology, Shanghai Institute of Applied Physics, Chinese Academy of Sciences, Shanghai 201800, China; School of Chemistry and Chemical Engineering, Frontiers Science Center for Transformative Molecules, and Shanghai Key Laboratory for Nucleic Acids Chemistry and Nanomedicine, Institute of Molecular Medicine, Renji Hospital, School of Medicine, Shanghai Jiao Tong University, Shanghai 200240, China

**Keywords:** synchrotron-based X-ray microscopy, cellular imaging, nanoscale protein localization, genetically encoded X-ray tags, 3,3^′^-diaminobenzidine polymers

## Abstract

Spatial resolution defines the physical limit of microscopes for probing biomolecular localization and interactions in cells. Whereas synchrotron-based X-ray microscopy (XRM) represents a unique approach for imaging a whole cell with nanoscale resolution due to its intrinsic nanoscale resolution and great penetration ability, existing approaches to label biomolecules rely on the use of exogenous tags that are multi-step and error-prone. Here, we repurpose engineered peroxidases as genetically encoded X-ray-sensitive tags (GXET) for site-specific labeling of protein-of-interest in mammalian cells. We find that 3,3^′^-diaminobenzidine (DAB) polymers that are in-situ catalytically formed by fusion-expressed peroxidases are visible under XRM. Using this new tag, we imaged the protein location associated with the alteration of a DNA-methylation pathway with an ultra-high resolution of 30 nanometers. Importantly, the excellent energy resolution of XRM enables multicolor imaging using different peroxidase tags. The development of GXET enlightens the way to nanoscopic imaging for biological studies.

## Teaser

We report the development of genetcially-encoded tags for X-ray microscopic (XRM) imaging, which allows nanoscale localization of proteins in cells.

## INTRODUCTION

Determining the localization of proteins using fluorescent microscopy has been integral to elucidating many cellular processes. The development of super-resolution (SR) fluorescence microscopy enables the monitoring of cellular functional biomolecules at 10- to 100-nm resolution [1,2], shedding new light on the nanoscale imaging of cellular events. Electron microscopy (EM) provides a powerful means to image cells at the atomic resolution [3]. Especially, recently developed genetically encoded EM tags, e.g. engineered ascorbate peroxidase (APEX2), have established a paradigm-shifting framework for labeling cellular targets for EM imaging with unprecedented resolution [4]. Nevertheless, the low penetration ability of electrons restricts the use of EM for whole-cell imaging [5].

Synchrotron-based X-ray microscopy (XRM) offers a unique approach to imaging whole cells in the sub-100-nm regime [6,7]. XRM is intrinsically a type of nanoscopy because of the very short wavelength of X-rays. Compared with electron beams, X-rays have a greater ability to penetrate biological specimens, hence intact cells can be imaged without specimen sectioning. Probably more importantly, X-rays provide excellent energy resolution that allows accurate spectral identification, which in principle allows the nanoscale cellular imaging of bio-targets with high specificity. However, currently available X-ray-sensitive probes generally exploit the use of high-atomic-number elements, e.g. metal or semiconductor nanoparticles (NPs). Staining cells with these exogenous labeling tags often leads to poor specificity due to cross reactions [8–10]. Hence, the development of X-ray-sensitive probes with high labeling specificity is highly desirable for cellular imaging.

For optical SR/EM microscopy, appropriate imaging tags have achieved great successes in gaining a better understanding of cellular processes. The advent of genetically encoded fluorescent proteins, such as the GFP-like family, has revolutionized cell biology using optical SR microscopy [11,12]. Newly developed genetically targetable EM tags, especially engineered ascorbate peroxidase (APEX2), provide unprecedented labeling techniques for imaging any cellular bio-targets using EM [4]. Inspired by the strong adsorption capacity for organic material in the X-ray-transmissive ‘water window’ [13] and the polymerization reaction after peroxidase treatment [3], here we repurpose engineered peroxidase for XRM.

## RESULTS

**Figure 1.**
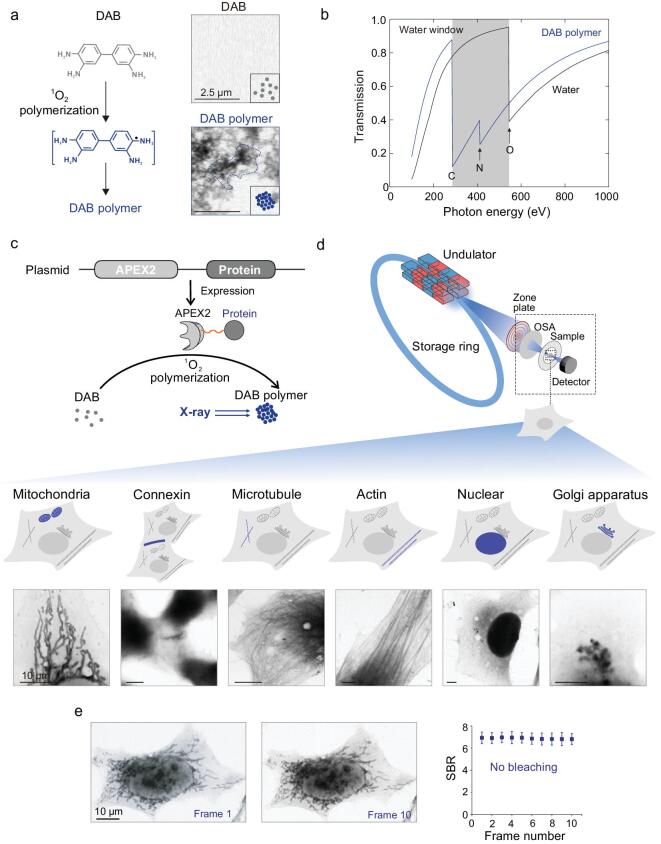
Repurpose-engineered peroxidase as genetically encoded tags for protein localization with XRM. (a) Schematic showing the catalytic polymerization of DAB into DAB polymer (left) and X-ray imaging of the DAB polymer (right). (b) X-ray-absorption spectra of water and DAB polymer. In the ‘water window’, absorption by carbon and nitrogen is much stronger than that by oxygen. (c) Schematic showing APEX2 as genetically encoded tags for protein localization with XRM. Through using fusion-expression plasmids including APEX2 and biotargets, they have high specificity. Following catalyzing the polymerization of DAB into localized X-ray-visible dense DAB polymers, they localize and image various cellular targets with high resolution. (d) STXM images of cellular proteins and specific amino-acid sequence: COX4 (mitochondrial), Cx43, α-tubulin, β-actin, NLS and GalT. Scale bars: 10 μm. (e) Photostability characterization of this genetically encoded tag for protein localization with XRM. No photo-bleaching occurred after 10 frames of STXM scans (for each STXM scan, the SBR of 10 loci was calculated and averaged to obtain a single value.). Scale bars: 10 μm.

We first examined whether the APEX2 system could be repurposed to provide contrast enhancement in XRM. APEX2 catalyses local polymerization of diaminobenzidine (DAB) [14], generating dense precipitates rich in carbon (C) and nitrogen (N). We analysed the X-ray absorption of DAB precipitates derived from an APEX2-catalysed reaction (Fig. 1a, left). We found that, within the X-ray-transmissive ‘water window’ (wavelength 2.4–4.4 nm, or photon-energy range ∼280–530 eV, between the K-shell absorption edges of C and O) typically used in bioimaging, the absorption of DAB precipitates is far stronger than that of water or soluble DAB monomers (Fig. 1b). By using synchrotron-based scanning-transmission X-ray microscopy (STXM) at a photo energy of 525 eV (2.36 nm wavelength within the water window), we observed that DAB aggregates with high contrast (dark-colored structures in Fig. 1a, right; Supplementary Figs 1 and 2). In comparison, DAB monomers in solution showed little contrast from the background (Fig. 1a, right). These results suggest that DAB can be used as an XRM contrast agent specific to APEX2 catalysation.

We proceeded to test the utility of the genetically encoded tagging system for X-ray imaging in cells (Fig. 1c). We constructed plasmid-expressing APEX2 fused to mitochondrial cytochrome c oxidase subunit 4 (APEX2–COX4) and transfected it into HeK293T cells. After DAB staining, the cell specimen was imaged by STXM with photon energies ranging from 395 to 550 eV (Supplementary Fig. 3). The resulting images show high-contrast (dark-colored) structures with typical shapes of mitochondria (Fig. 1d and Supplementary Fig. 3). We found that the optimal resolution (measured by the full width at half maximum, or FWHM, of a filament structure) and signal-to-background ratio (SBR) could be obtained at 525 eV. Here, although untagged cell structures containing C and N also generated contrast in this energy range (which lays the basis for unlabeled XRM cell imaging), the DAB precipitates can produce much stronger contrast (3.1–8.1-fold enhancement) compared to the former, thus allowing us to specifically track the functional molecules of interest under XRM via the genetic tagging.

To demonstrate the generality of this tagging system, we next sought to tag and image diverse subcellular structures. We separately transfected cells with APEX2-fused connexin-43, α-tubulin, β-actin, nuclear localization sequence (NLS) and galactosyltransferase (GalT, a Golgi marker). Correspondingly, their XRM images (Fig. 1d and Supplementary Fig. 4) revealed high-contrast structures with typical shapes of gap junctions, microtubules, microfilaments, cell nucleus and Golgi apparatus, in agreement with fluorescent microscopic images [15–19]. In the contrary, cells free of tag labeling showed no such high-contrast structures under STXM (Supplementary Figs 5 and 6). These results suggest that the APEX2 tagging system can be used to specifically tag diverse peptides or proteins in cells.

Next, we evaluated the photostability of the X-ray tag under XRM. After 10 continuous frames of STXM scans of the tag-labeled mitochondria samples, we observed little change in the SBR of the tagged structures, suggesting good photostability of the tag system under XRM (Fig. 1e and Supplementary Fig. 7). In comparison, the sample labeled with endogenous fluorescent tags showed a decrease in the fluorescence intensity of up to 28% under confocal microscopy in the same number of frames (Supplementary Fig. 8). The photostability of this tag allows repetitive X-ray scanning with high density, and thus enables us to obtain images with high spatial and energy resolution.

**Figure 2.**
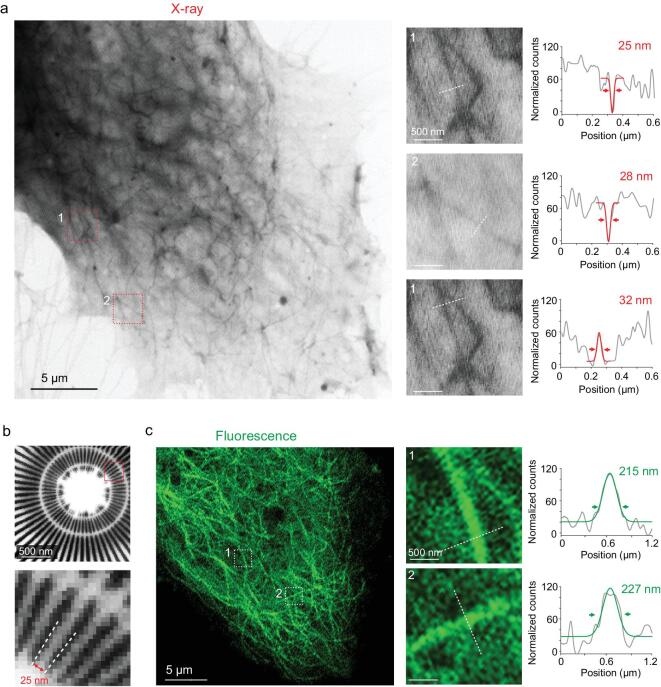
Super-high-resolution X-ray imaging of tubulin by using genetically encoded tags. (a) HeLa cells were transfected with APEX2–tubulin plasmids and then dyed with DAB. Left: X-ray images of α-tubulin in cells. Scale bar: 5 μm. Right: magnification of the boxed regions and the intensity profile of a single fibril or fibril crossing along dashed lines. Scale bars: 500 nm. (b) X-ray imaging of test star-pattern sample. The inner circle demonstrates a spatial pattern of 25 nm. Scale bar: 500 nm. (c) HeLa cells were transfected with EGFP–tubulin plasmids. Left: conventional confocal images of tubulin in cells. Scale bar: 5 μm. Right: magnification of the boxed regions and the intensity profile of a single fibril along dashed lines. Scale bars: 500 nm. Each intensity profile was acquired by loading the gray value of the image using ImageJ. Structural features were inferred with 1D Gaussian fits and the FWHM values are shown on each profile.

We then quantified the spatial resolution of the XRM cell imaging based on this tagging system. The optimal resolution of our XRM system was ∼25 nm (determined with the imaging of a star test pattern shown in Fig. 2b, with a scanning step of 15 nm). We imaged the tagged microtubule filaments in cells using this system (Fig. 2a, left). Line profiles of the filaments indicate that their representative FWHM was about 25–28 nm (Fig. 2a, right). Moreover, two adjacent filaments with a peak-to-peak distance down to ∼30 nm could be clearly distinguished, suggesting the nanoscale spatial resolution of our system (Fig. 2a, right). In contrast, under confocal microscopy, the filaments tagged with enhanced green fluorescent protein (EGFP)-tubulin showed a typical FWHM value of >200 nm (Fig. 2c), consistently with the theoretical resolution limit of optical microscopy (∼200 nm) due to the diffraction limit. These data suggest that our tagging system in combination with XRM allows a nanoscale spatial resolution (∼30 nm), which is approximately an order of magnitude improved compared with classic optical microscopy. We also envisaged the development of our tagging system, such as enhanced APEX2 with improved activity toward DAB, in the hope that faster kinetics could reduce the reaction time and minimize the diffusion of DAB precipitates within cells, thus further improving the spatial resolution down to a few nanometers, which provides unprecedented power for cellular imaging.

**Figure 3.**
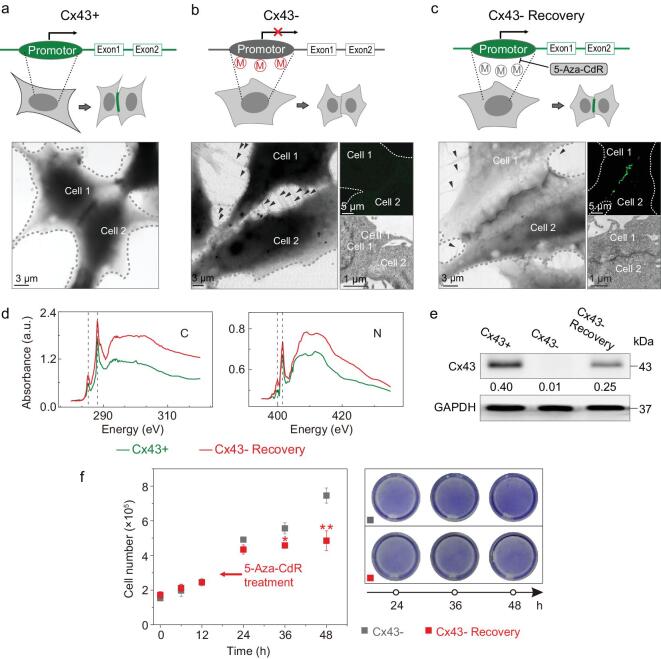
Tracking of the regulation of Cx43 expression by DNA methylation in cells with genetically encoded X-ray tags. For Hek293T cells (Cx43+), they were transfected with Cx43–GFP–APEX2 plasmids; for HeLa cells (Cx43– or Cx43– Recovery), they were treated without or with methylation inhibitor 5-Aza-CdR and then transfected with Cx43–GFP–APEX2 plasmids and dyed with DAB. (a–c) Cx43+ (a); 43– (b); Cx43– Recovery (c). Upper: schematic showing the regulation of Cx43 expression by DNA methylation in cells. Lower: X-ray images of Cx43 in cells. Black arrows indicate the primary cilia on the surface of HeLa cells. Scale bars: 3 μm. Inset: confocal and transmission electron microscope (TEM) images of HeLa cells. Scale bars: 5 μm (confocal) and 1 μm (TEM). (d) NEXAFS spectra analysis of the boxed regions in (a), middle. Dashed lines indicate the main adsorption features of the peptide bonds. (e) Immunoblots of Cx43 in treated cells. Glyceraldehyde 3-phosphate dehydrogenase (GAPDH) was used as the loading control. (f) Growth curves of and crystal-violet-staining analysis-treated HeLa cells. ^*^P < 0.05; ^**^P < 0.01, significantly different from Cx43–.

Following this proof of concept, we sought to apply this tagging system in visualizing the change in intercellular connections associated with the DNA-methylation pathway. Intercellular connections and channels (e.g. gap junctions) allow intercellular communication between cells to regulate cell death, proliferation, differentiation and oncogenesis [20,21]. A high level of methylation has been reported to occur in the promoter region of connexin-43 (Cx43, a component protein of gap junctions) in diverse tumor cells [22] (Supplementary Fig. 9), suggesting the important role of DNA methylation in regulating intercellular connections and tumor-cell growth [23,24]. Thus, direct visualization of subtle changes in the intercellular-connection structures can provide a powerful means to help understand the mechanism, which however remains challenging due to the resolution limit of optical microscopy [3]. Here, we set out to tag Cx43 using our system. We constructed a plasmid with the fusion protein Cx43–GFP–APEX2 and transfected it into a normal cell line Hek293T (known as Cx43 positive) and a carcinoma cell line HeLa (known as Cx43 repressed), respectively. Under XRM, we observed high-contrast, continuous stripes at the boundaries between adjacent Hek293T cells (Fig. 3a and Supplementary Figs 11a and 12), which can be confirmed by fluorescent microscopy (Supplementary Fig. 10), suggesting successful visualization of Cx43 and gap junctions. In comparison, these continuous stripes could not be found between HeLa cells (Fig. 3b and Supplementary Figs 11b and 12), in agreement with the low level of Cx43 fluorescence under confocal microscopy, suggesting the suppression of Cx43 in HeLa cells. Instead, we observed many ciliary connections (marked with arrows in Fig. 3b and Supplementary Fig. 11b) between the cells under XRM. These primary cilia have been reported to be crucial for the development of cancers [25]. Notably, these refined intercellular structures can hardly be visualized by confocal microscopy or EM (Fig. 3b, inset). To verify the dependency of the intercellular connections on Cx43 methylation, we further treated the HeLa cells with the methylation inhibitor 5-aza-2^′^-deoxycytidine (5-Aza-CdR) (known as Cx43 recovery). As a result, we observed the appearance of dark stripes between the methyltransferase-inhibited HeLa cells (Fig. 3c and Supplementary Figs 11c and 12), indicating the formation of gap junctions similar to those in Cx43-positive cells. Meanwhile, the number of primary cilia between the methyltransferase-inhibited cells was largely reduced (Fig. 3c and Supplementary Fig. 11c). Further, near-edge X-ray-absorption fine-structure (NEXAFS) spectra and immunoblotting of Cx43 proteins in these two cell lines also confirmed the recovery of Cx43 expression in methyltransferase-inhibited HeLa cells (Fig. 3d and e, and Supplementary Fig. 13). By cell counting and crystal-violet-staining analysis, we also found that the growth rate of the methyltransferase-inhibited HeLa cells is significantly slower than that of untreated HeLa cells (Fig. 3f). These data confirmed that the methylation of Cx43 is closely related to the status of the intercellular connections and communications. Our genetically encoded tagging system in cooperation with XRM enables the visualization of details in these biological pathways with nanoscale spatial resolution.

**Figure 4.**
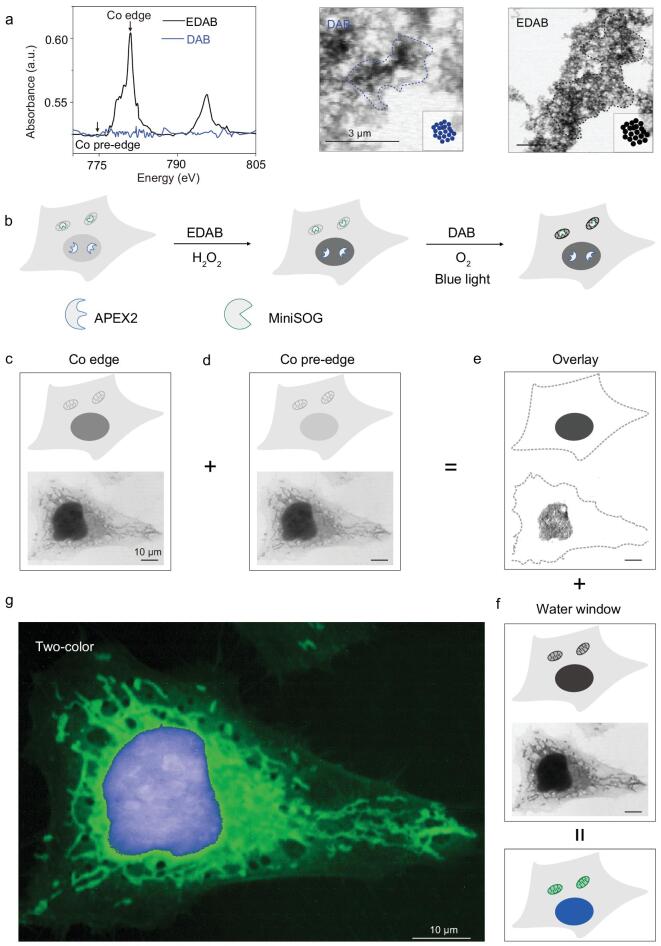
Multicolor X-ray microscopy for simultaneous visualization of two bio-targets. (a) NEXAFS spectra analysis and X-ray images of APEX2-catalysed different substrate molecule polymers *in vitro*. Arrows indicate the energies above and below the Co-absorption edge (780.68 and 775 eV, respectively). Scale bars: 3 μm. (b–g) HeLa cells were co-transfected with NLS–APEX2 and mito-miniSOG plasmids, and stained with EDAB and DAB in turn. Schematic showing (upper) and experimental (lower) of multicolor X-ray microscopy for simultaneous visualization of cell nuclei and mitochondria. Scale bars: 10 μm. Pseudocolored images of the two-hue representative bio-targets with nanoscale resolution were generated using ImageJ. Scale bar: 10 μm.

Finally, we demonstrate XRM dual-color imaging of cells by introducing different peroxidase tags (Fig. 4b depicts our design). Due to the excellent energy resolution, XRM can implement multicolor imaging by introducing elements with distinguishable adsorption energies. Here, we introduced enhanced DAB (EDAB) as another substrate (in addition to DAB), which contains Co element and possesses characteristic adsorptions at 794 and 779 eV in the NEXAFS spectrum, and thus it can be discriminated from DAB, providing another ‘color’ under XRM (Fig. 4a). To tag different cellular structures, we introduced the photosensitizer miniSOG (mini singlet oxygen generator) as another genetically encoded peroxidase tag (in addition to the APEX2 tag), which can be photoactivated by blue light [26]. For dual-color imaging, HeLa cells were transfected with plasmids carrying NLS–APEX2 (targeting cell nucleus) and mito-miniSOG (targeting mitochondria). For the first round of staining, EDAB was added to allow polymerization catalysed by APEX2. Subsequently, the specimen was stained with DAB under a stream of pure oxygen, allowing polymerization catalysed by miniSOG under the blue light (Fig. 4b). For cell nucleus tagged with APEX2–EDAB, two STXM absorption images with energies above (780 eV) and below (775 eV) the Co-absorption edge were acquired separately and processed with a dual-energy ratio-contrast processing method [27] (Fig. 4c–e). The resulting image shows the region of the cell nucleus with high contrast, which can be attributed to the precipitation of Co-enriched EDAB there. For mitochondria tagged with miniSOG-DAB, we acquired the image at 525 eV in the water window (Fig. 4f), which clearly shows typical structures of mitochondria. In this image, the cell-nucleus region also presents high intrinsic contrast. However, by overlaying it with the processed image of the cell nucleus, we obtained a dual-color image in which the cell nucleus and mitochondria can be well distinguished (Fig. 4g). To demonstrate the generality of this dual-color tagging system, we next transfected HeLa cells with plasmids carrying Golgi–APEX2 (targeting Golgi) and mito-miniSOG (targeting mitochondria). Then, cell samples were treated by the same method as mentioned above, and we obtained a dual-color image in which the Golgi and mitochondria can be well distinguished (Supplementary Fig. 14). These results suggest the potential of this tagging system for multiplexed XRM imaging. Given the high energy resolution of XRM, the multiplexability can be readily expanded by introducing more elements.

## DISCUSSION AND CONCLUSION

In summary, we repurposed engineered peroxidases as genetically encoded tags for nanoscale protein localization with XRM. By using this tagging system, we obtained images of diverse molecules and structures in cells with a spatial resolution of ∼30 nm. We visualized the change in intercellular connections among tumor cells dependent on DNA methylation. We also demonstrate the dual-color imaging of cellular structures utilizing the high energy resolution of XRM. This tagging system shows several distinct features: (i) This technique generates XRM contrast based on a specific chemical reaction (i.e. DAB polymerization), which is well restricted in a nanometer-scale area, and thus can provide nanoscale precision in target locating. In comparison, for transmission electron microscope (TEM) tags, the global OsO_4_ staining used to provide high contrast (high electron density) is a process of physical diffusion and adsorption [3,28], which may cause nonspecific staining that compromises the SBR and locating precision (Supplementary Fig. 13) [29]. (ii) Compared to exogenous probes, which work temporally, genetically encoded tags can be maintained across cell-division cycles without signal loss, and thus can be used to track the fate of target molecules across cell generations. Besides, the exogenous probe-labeling process may disturb cellular ultrastructures due to the permeabilizing treatment [30], resulting in distortions in target locating. This disadvantage can be avoided by the endogenous, genetically encoded tags. (iii) Compared to fluorescent tags, the X-ray tags show high photostability, enabling repetitive X-ray scanning with high pixel density, high penetration and multiple energy ranges, which in principle allows XRM imaging with ultra-high energy resolution and spatial resolution in thick samples (e.g. whole cells or tissues). In future, we will be devoted to the development of a spectrum of X-ray tags in combination with orthogonal substrate molecules, which would allow high-multiplexed XRM imaging for targeting multiple molecules and structures in cells.

## METHODS

### Plasmid construction

The pcDNA3 rat connexin43 (Cx43)–GFP–APEX2, mito-V5–APEX2 (named mito–APEX2 in text), APEX2–tubulin and APEX2–actin (Addgene plasmid # 49385, 72480, 66171, 66172) were gifts from Alice Ting. Cx43 human cDNA was synthesized, amplified and inserted into pcDNA3 rat Cx43–GFP–APEX2 by standard restriction cloning methods to create pcDNA3 human Cx43–GFP–APEX2. DNA-encoding human galactosyltransferase (GalT)–APEX2 was synthesized and cloned into pcDNA3.1 vector to generate Golgi–APEX2. The APEX2 sequence in mito–APEX2 was replaced by the EGFP gene sequence to generate mito-EGFP. EGFP–Tubulin-6 was a gift from Michael Davidson (Addgene plasmid # 56450).

DNA-encoding NLS–APEX2 was synthesized by overlap extension polymerase chain reaction (PCR) and cloned into pcDNA3 vector. MiniSOG-Mito-7 (named mito-miniSOG in text) was a gift from Michael Davidson (Addgene plasmid # 57773).

### Preparation of X-ray-sensitive genetic probes

HEK 293T cells were grown in the Dulbecco's modified Eagle's medium (DMEM, Gibco) with 10% fetal bovine serum (FBS) and antibiotics (100 units/mL penicillin and 100 μg/mL streptomycin). HeLa cells were grown in the minimal essential medium (MEM, Gibco) with 10% FBS and antibiotics. All cells were kept at 37°C under 5% CO_2_.

The Si_3_N_4_ windows were previously put into 24-well plates and sterilized by ultraviolet rays. A cell suspension (2 × 10^5^ cells/mL for Hek293T and 7 × 10^4^ cells/mL for HeLa) was dispensed into 24-well plates and incubated overnight to allow cell adherence to the Si_3_N_4_ windows. After 24-h transfection with plasmids as required: hCx43–GFP–APEX2, APEX2–EGFP–tubulin, mito-V5–APEX2, APEX2–actin, NLS–EGFP–APEX2 and Golgi–APEX2 plasmids with the v/v/w of lipo3000/p3000/plasmids was 0.75 μL : 1μL : 500 ng, cells were fixed on ice using chilled 2% glutaraldehyde (SPI-CHEM) in Dulbecco's phosphate-buffered saline (DPBS) buffer containing Mg^2+^ for 40 min. After being washed twice in chilled DPBS, they were treated by 20 mM glycine in DPBS for 5 min to quench unreacted glutaraldehyde. Then, cells were washed again and stained with freshly diluted DAB (1.4 mM, Sigma) and H_2_O_2_ (0.03% v/v) in chilled DPBS for 1 min (for most plasmids) or 1 h (for α-tubulin) as required. Finally, cells were fixed with a little 4% paraformaldehyde in DPBS and then dehydrated in a graded gradient ethanol series.

### Synchrotron-based XRM and NEXAFS spectra analysis

The absorption and differential phase contrast images were obtained by STXM. STXM measurements were performed at the 10ID-1 Soft X-ray Spectromicroscopy beamline of the Canadian Light Source (CLS), the BL08U1-A Soft X-ray Spectromicroscopy beamline of the Shanghai Synchrotron Radiation Facility (SSRF) and the 10A Soft X-ray Nanoscopy beamline of the Pohang Light Source (PLS) II. The beamlines are equipped with a 25-nm outermost-zone zone plate (CXRO, Berkeley Lab). The monochromatic X-ray beam was focused to a 30-nm spot on the sample. The samples on a transparent Si_3_N_4_ window were raster scanned by a piezoelectric stage with synchronized detection of transmitted X-rays to generate the images. The STXM chamber was filled with Helium (CLS, PLS) or maintained in a vacuum (SSRF). Images were recorded within the energy region of the *K*-edges of C (between 280 and 320 eV), N (between 395 and 435 eV) and O (between 525 and 560 eV) and the *L*-edge of Co (between 770 and 810 eV). C-, N- and Co-NEXAFS spectra were obtained for every 0.15- to 1-eV step, while O-NEXAFS was obtained for every 0.2- to 1-eV step. The extraction of images and spectra from energy-dependent image data was conducted using Xis2000 software (A. P. Hitchcock, the aXis2000 analysis package is written in an Interactive Data Language [IDL], http://unicorn.mcmaster.ca/aXis2000.html). NEXAFS spectra from each interested region and from the control region were extracted from the image stacks using an image mask, which allows only selecting the region(s) of interest.

### Analysis of the regulation of Cx43 expression by DNA methylation in cells

A Hek293T cell line (Cx43+) was transfected with hCx43–GFP–APEX2 plasmids for 24 h. The HeLa cell line was treated with or without 50-μM methylation inhibitor 5-aza-CdR for 24 h and then transfected with hCx43–GFP–APEX2 plasmid for 48 h. All cells were fixed and stained with DAB by the same method as mentioned above.

### Two-color XRM

The HeLa cell suspension (7 × 10^4^ cells/mL) was dispensed into 24-well plates and incubated overnight. After 24-h co-transfection with NLS–APEX2 (or Golgi–APEX2) and mito-miniSOG plasmids at 80% confluence with the v/v/w/w of lipo3000/p3000/NLS–APEX2 (or Golgi–APEX2)/mito-miniSOG was 1.5 μL : 2 uL : 500 ng : 500 ng, cells were fixed on ice using chilled 2% glutaraldehyde in DPBS buffer containing Mg^2+^ for 40 min. Then, cells were stained with freshly prepared EDAB (0.2 mg/mL DAB, 1 mg/mL CoCl_2_) to initial APEX2 depositing EDAB polymer. Following five 2-min washes with phosphate buffer saline (PBS), cells were rinsed for 5 min three times with fresh 100 mM acetylimidazole in 0.15 M NaCl to block further polymerization of EDAB. For the subsequent photo-oxidation reaction, freshly prepared DAB solution (0.2 mg/mL) was placed on ice and added to the cells. A stream of pure oxygen was gently blown continuously over the top of the solution. Then, cells were illuminated to excite miniSOG depositing DAB polymer using a 473-nm blue laser for 15 min. Cells were rinsed, fixed and dehydrated by the same method as described above.

The absorption and differential phase contrast images were obtained by STXM at the 10A Soft X-ray Nanoscopy beamline of the Pohang Light Source (PLS) II. The beamlines are equipped with a 40-nm outermost-zone zone plate (CXRO, Berkeley Lab). The monochromatic X-ray beam was focused to a 50-nm spot on the sample. The samples on a transparent Si_3_N_4_ window were raster scanned by a piezoelectric stage with synchronized detection of transmitted X-rays to generate the images. Three photon energies were chosen, namely E1 = 780.68 eV, E2 = 775 eV and E3 = 525 eV, to scan the cell sample pixel by pixel. E1 and E2 are just above and below the absorption edges of cobalt and a dual-energy ratio-contrast imaging method [27] was applied to obtain the overlay of the absorption-contrast images of the cobalt-distribution images. After overlaying the STXM imaging at 525 eV (in the region of the ‘water window’), two bio-target distribution images were obtained. Pseudocolored images of the two-hue representative bio-targets were generated using ImageJ.

## Supplementary Material

nwaa055_Supplemental_File
