# Supplementary material for: Genetically encoded X-ray cellular imaging for nanoscale protein localization
Source: Natl Sci Rev. 2020 Apr 2;7(7):1218–27. doi: 10.1093/nsr/nwaa055 (PMC8288996; doi:10.1093/nsr/nwaa055)
Supplement: nwaa055_Supplemental_File [file nwaa055_supplemental_file.docx]

**Supplementary Information**

**Genetically encoded X-ray cellular imaging for nanoscale protein localization**

Huating Kong^1,2,‡^, Jichao Zhang^1,2,‡^, Jiang Li^1,2,‡^, Jian Wang^3^, Hyun-Joon Shin^4^, Renzhong Tai^1^, Qinglong Yan^2^, Kai Xia^2^, Jun Hu^1,2^, Lihua Wang^1,2^, Ying Zhu^1,2^*, Chunhai Fan^5^*

^1^Bioimaging Center, Shanghai Synchrotron Radiation Facility, Zhangjiang Laboratory, Shanghai Advanced Research Institute, Chinese Academy of Sciences, Shanghai 201210, China

^2^Division of Physical Biology, CAS Key Laboratory of Interfacial Physics and Technology, Shanghai Institute of Applied Physics, Chinese Academy of Sciences, Shanghai 201800, China

^3^Canadian Light Source Inc., University of Saskatchewan, Saskatoon, SK S7N 2V3, Canada.

^4^Pohang Accelerator Laboratory, POSTECH, Pohang, 37673, Republic of Korea.

^5^School of Chemistry and Chemical Engineering, Frontiers Science Center for Transformative Molecules, and Shanghai Key Laboratory for Nucleic Acids Chemistry and Nanomedicine, Institute of Molecular Medicine, Renji Hospital, School of Medicine, Shanghai Jiao Tong University, Shanghai 200240, China

***Corresponding author**. E-mail: zhuying@zjlab.org.cn; fanchunhai@sjtu.edu.cn

^‡^Equally contributed to this work.

**Supporting text.**

**Expression and purification of APEX2**

pTRC-APEX2 was a gift from Alice Ting (Addgene plasmid #72558). The plasmid was transformed into competent *Escherichia coli* BL21 (DE3) cells. Individual colonies were amplified in 1 L Luria broth (LB) supplemented with 420 µM isopropyl β-d-1- thiogalactopyranoside (IPTG) as inducing agent and 1 mM 5-aminolevulinic acid hydrochloride (Sigma) at room temperature overnight in the dark [[1](#_ENREF_1)]. APEX2 proteins were purified from the supernatant using a Ni-NTA agarose affinity column (Qiagen). The purified APEX2 was characterized by SDS-PAGE.

**APEX2-catalyzed polymerization of DAB (EDAB) in vitro**

DAB or metal enhanced DAB (EDAB) aqueous solution and H_2_O_2_ were added to the PBS of pH7.4, respectively. The final concentration of DAB or EDAB was 0.4 mg/mL and that of H_2_O_2_ was 10 mM. APEX2 proteins with final concentration of 100 nM were added into the mixed solution mentioned above and vortexed thoroughly for 15 min to generate X-ray visible DAB or EDAB polymer.

**Fluorescence microscopy**

Confocal laser scanning microscopy was carried out with a Leica TCS sp8 microscope equipped with a multi-line argon laser and a HC×PL APO 63×, 1.4 NA oil-immersion objective.

Cells for imaging were grown in 35 mm confocal dishes at a density of 2×10^5^ cells/well (for Hek293T) or 7×10^4^ cells/well (for HeLa) and incubated overnight to allow for cell adherence. Hek293T cells were transfected with Cx43-GFP-APEX2 or hCx43-GFP-APEX2 plasmids. HeLa cells were transfected with EGFP-Tubulin-6 or mito-EGFP plasmids. The v/v/w of lipo3000/p3000/plasmids for all cells was 0.75 μL: 1μL: 500 ng. After 24 h transfection, observations were done by confocal microscopy (λ_ex_ 488 nm, λ_em_ 500-550 nm).

**Transmission electron microscopy**

HeLa cell line was treated with methylation inhibitor 5-aza-CdR and transfected with hCx43-GFP-APEX2 plasmid by the same method as described in text (Method section “Analysis of regulation of Cx43 expression by DNA methylation in cells”). After fixation with 2% glutaraldehyde for 40 min, cells were treated for 5 min in chilled sodium cacodylate buffer containing 20 mM glycine to quench unreacted glutaraldehyde, and then stained with freshly diluted DAB (1.4 mM) and H_2_O_2_ (0.03%) in chilled buffer for 1 min. Following twice wash in chilled buffer, cells were stained with 2% OsO_4_ on ice for 1 h, dehydrated and then embedded in Epon618. Ultrathin sections of the embedded cells were examined by transmission electron microscope (TEM, JEOL-1230; JEOL).

**Western blotting analysis**

After treatment, cells were washed twice with PBS and harvested by SDS-loading sample buffer. Protein samples were analyzed by 15% SDS-PAGE and blotted to PVDF membranes. The blots were blocked for 1 h using 6% nonfat milk in PBST (PBS containing 0.1% Tween20) buffer and then incubated overnight at 4°C with the primary antibodies as required: anti-connexin 43 (1:1000 dilution, Sigma) or glyceraldehyde 3-phosphate dehydrogenase (GAPDH) (1:1000 dilution, Abcam). After extensive washing, the blots were probed with a goat anti-rabbit horseradish peroxidase-conjugated antibody (1:10000 dilution, KPL) for 1 h. The blots were then developed by incubation with chemiluminescence (ECL) plus and exposed to X-ray film. The expression of GAPDH was used as the protein loading control.

**Cell growth curve drawing**

HeLa cells for growth curve drawing were grown in 12-well plates at a density of 7×10^4^ cells/well. After treatment, cell number at each timepoint was determined by an automated cell counter (Countstar). All data was based on three independent measurements.

**Crystal violet assay**

HeLa cells for crystal violet assay were grown in 24-well plates at a density of 7×10^4^ cells/well. After treatment, cells were fixed with 4% paraformaldehyde for 10 min, washed 2 × 2 min with millipore water and stained with 0.1% crystal violet (Beyotime) for 10 min. Following 5×2 min wash with millipore water, cell plates were dried 12 h at room temperature for imaging.

**Preparation of electron beam-sensitive genetic probes**

Hek293T cells were cultured, transfected with Cx43-GFP-APEX2 plasmid and stained with DAB by the same method as described in text (Method section “Preparation of X-ray-sensitive genetic probes”). After twice wash in chilled sodium cacodylate buffer, cells were stained with 2% OsO_4_ in DPBS on ice for 1 h. Following fixation with a few of 4% paraformaldehyde in DPBS, cells were dehydrated in a graded gradient ethanol series and observed under STXM for comparison with the X-ray-sensitive genetic probes.

**Statistical analysis**

Unless otherwise stated, all results are expressed as the mean ± standard deviation from three replicate experiments conducted in a parallel manner. Statistical significance of the data was determined using t-tests or one-way analysis of variance (ANOVA) using SPSS 19. ** equals P< 0.01; * equals P < 0.05.

**Supplementary figures and tables.**

|  | **Rat Cx43** | **Human Cx43** |
| --- | --- | --- |
| **AA sequence** | mgdwsalgklldkvqaystaggkvwlsvlfifrilllgtavesawgdeqsafrcntqqpgcenvcydksfpishvrfwvlqiifvsvptllylahvfyvmrkeeklnkkeeelkvaqtdgvnvemhlkqieikkfkygieehgkvkmrggllrtyiisilfksvfevaflliqwyiygfslsavytckrdpcphqvdcflsrptektifiifmlvvslvslalniielfyvffkgvkdrvkgrsdpyhattgplspskdcgspkyayfngcssptaplspmsppgyklvtgdrnnsscrnynkqaseqnwanysaeqnrmgqagstisnshaqpfdfpddnqnakkvaaghelqplaivdqrpssrassrassrprpddlei | mgdwsalgklldkvqaystaggkvwlsvlfifrilllgtavesawgdeqsafrcntqqpgcenvcydksfpishvrfwvlqiifvsvptllylahvfyvmrkeeklnkkeeelkvaqtdgvnvdmhlkqieikkfkygieehgkvkmrggllrtyiisilfksifevaflliqwyiygfslsavytckrdpcphqvdcflsrptektifiifmlvvslvslalniielfyvffkgvkdrvkgksdpyhatsgalspakdcgsqkyayfngcssptaplspmsppgyklvtgdrnnsscrnynkqaseqnwanysaeqnrmgqagstisnshaqpfdfpddnqnskklaaghelqplaivdqrpssrassrassrprpddlei |

**Supplementary Table 1.** **Amino acid (AA) sequences of rat and human Cx43**. Different AA residues in rat and human Cx43 are shown in green and red colors, respectively.

**Supplementary Table 2.** **DNA sequences of rat and human cx43 gene cDNA.** DNA sequences encoding different AA of rat and human Cx43 are shown in green and red colors, respectively.

|  | **Rat Cx43** | **Human Cx43** |
| --- | --- | --- |
| **DNA sequence** | ATGGGTGACTGGAGTGCCTTGGGGAAATTACTGGACAAGGTCCAAGCCTACTCCACCGCTGGAGGGAAGGTGTGGCTGTCAGTGCTCTTCATATTCAGAATCCTGCTCCTGGGGACAGCTGTTGAGTCAGCTTGGGGTGATGAACAGTCTGCCTTTCGCTGTAACACTCAACAACCTGGCTGCGAAAACGTCTGCTATGACAAGTCCTTCCCCATCTCTCACGTGCGCTTCTGGGTCCTTCAGATCATATTCGTGTCTGTGCCCACACTCCTGTACTTGGCCCATGTGTTCTATGTGATGAGGAAGGAAGAGAAGCTAAACAAGAAAGAAGAGGAGCTCAAAGTGGCCCAGACTGACGGGGTCAACGTGGAGATGCACCTGAAGCAGATTGAAATCAAGAAGTTCAAGTACGGGATTGAAGAGCACGGCAAGGTGAAAATGAGGGGCGGCTTGCTGAGAACCTACATCATCAGCATCCTCTTCAAGTCTGTCTTCGAGGTGGCCTTCCTGCTCATCCAGTGGTACATCTATGGGTTCAGCTTGAGCGCGGTCTACACCTGCAAGAGAGATCCCTGCCCCCACCAGGTAGACTGCTTCCTCTCACGTCCCACGGAGAAAACCATCTTCATCATCTTCATGCTGGTGGTGTCCTTGGTGTCTCTCGCTTTGAACATCATTGAGCTCTTCTACGTCTTCTTCAAGGGCGTTAAGGATCGCGTGAAGGGAAGAAGCGATCCTTACCACGCCACCACTGGCCCACTGAGCCCATCAAAAGACTGCGGATCTCCAAAATACGCCTACTTCAATGGCTGCTCCTCACCAACGGCTCCACTCTCGCCTATGTCTCCTCCTGGGTACAAGCTGGTTACTGGTGACAGAAACAATTCCTCGTGCCGCAATTACAACAAGCAAGCTAGCGAGCAAAACTGGGCGAACTACAGCGCAGAGCAAAATCGCATGGGGCAGGCCGGAAGCACCATCTCCAACTCGCACGCCCAGCCGTTCGATTTCCCCGACGACAACCAGAATGCCAAAAAAGTTGCTGCTGGACATGAACTCCAGCCATTAGCCATCGTGGACCAACGACCTTCCAGCAGAGCCAGCAGCCGCGCCAGCAGCAGGCCTCGGCCTGATGACCTGGAGATT | ATGGGTGACTGGAGCGCCTTAGGCAAACTCCTTGACAAGGTTCAAGCCTACTCAACTGCTGGAGGGAAGGTGTGGCTGTCAGTACTTTTCATTTTCCGAATCCTGCTGCTGGGGACAGCGGTTGAGTCAGCCTGGGGAGATGAGCAGTCTGCCTTTCGTTGTAACACTCAGCAACCTGGTTGTGAAAATGTCTGCTATGACAAGTCTTTCCCAATCTCTCATGTGCGCTTCTGGGTCCTGCAGATCATATTTGTGTCTGTACCCACACTCTTGTACCTGGCTCATGTGTTCTATGTGATGCGAAAGGAAGAGAAACTGAACAAGAAAGAGGAAGAACTCAAGGTTGCCCAAACTGATGGTGTCAATGTGGACATGCACTTGAAGCAGATTGAGATAAAGAAGTTCAAGTACGGTATTGAAGAGCATGGTAAGGTGAAAATGCGAGGGGGGTTGCTGCGAACCTACATCATCAGTATCCTCTTCAAGTCTATCTTTGAGGTGGCCTTCTTGCTGATCCAGTGGTACATCTATGGATTCAGCTTGAGTGCTGTTTACACTTGCAAAAGAGATCCCTGCCCACATCAGGTGGACTGTTTCCTCTCTCGCCCCACGGAGAAAACCATCTTCATCATCTTCATGCTGGTGGTGTCCTTGGTGTCCCTGGCCTTGAATATCATTGAACTCTTCTATGTTTTCTTCAAGGGCGTTAAGGATCGGGTTAAGGGAAAGAGCGACCCTTACCATGCGACCAGTGGTGCGCTGAGCCCTGCCAAAGACTGTGGGTCTCAAAAATATGCTTATTTCAATGGCTGCTCCTCACCAACCGCTCCCCTCTCGCCTATGTCTCCTCCTGGGTACAAGCTGGTTACTGGCGACAGAAACAATTCTTCTTGCCGCAATTACAACAAGCAAGCAAGTGAGCAAAACTGGGCTAATTACAGTGCAGAACAAAATCGAATGGGGCAGGCGGGAAGCACCATCTCTAACTCCCATGCACAGCCTTTTGATTTCCCCGATGATAACCAGAATTCTAAAAAACTAGCTGCTGGACATGAATTACAGCCACTAGCCATTGTGGACCAGCGACCTTCAAGCAGAGCCAGCAGTCGTGCCAGCAGCAGACCTCGGCCTGATGACCTGGAGATC |


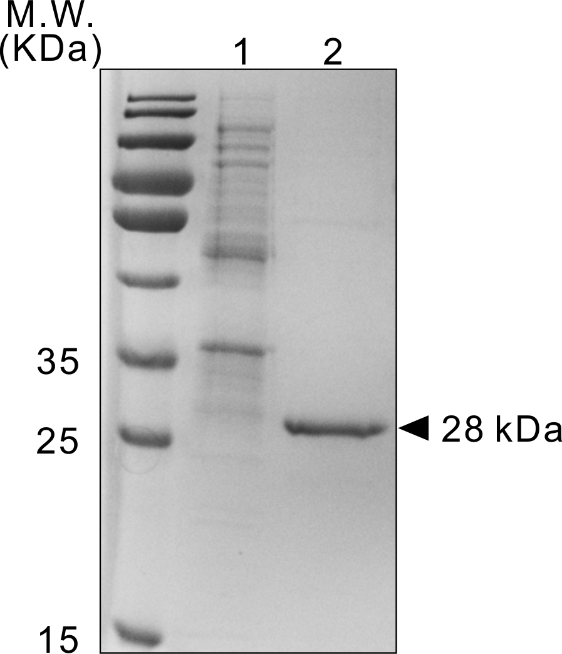


**Supplementary Figure 1. SDS-PAGE of E. coli (BL21) total protein extracts (lane 1) and purified APEX2 (lane 2)**. The purified APEX2 showed molecular weight of 28 kDa.


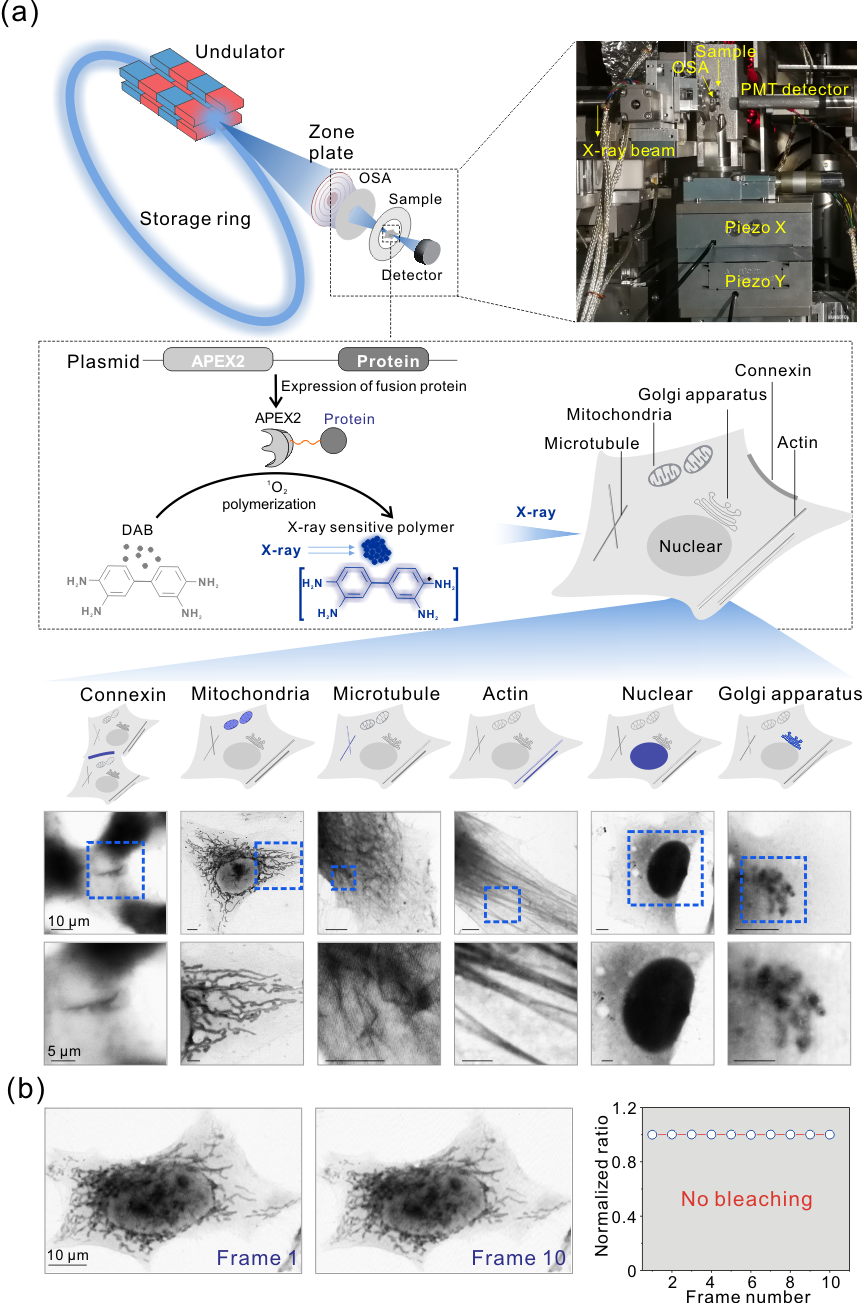


**Supplementary Figure 2. Physical map of the soft X-ray spectromicroscopy beamline.**


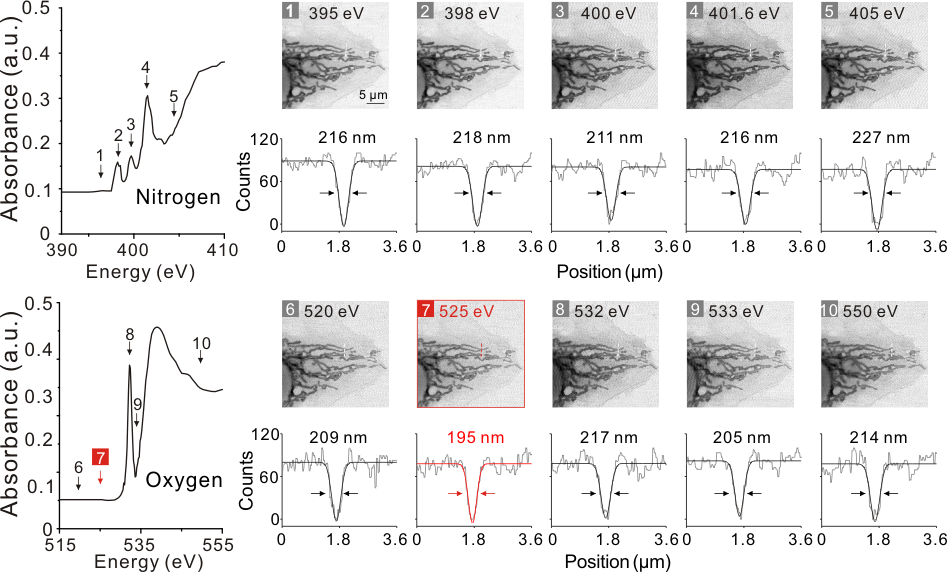


**Supplementary Figure 3. Energy optimization for X-ray imaging of cellular targets by using genetically-encoded tags.** HeLa cells were transfected with mito-APEX2 plasmids, and then dyed by DAB. X-ray images of mitochondria in cells under different energies. These energies were chosen around the "water window" soft X-ray region (around the *K*-edges of N and O). Each intensity profile was acquired by loading gray value of image using ImageJ. Structural features were inferred with one-dimensional Gaussian fits and FWHM values were shown on each profile. Scale bar: 5 μm.


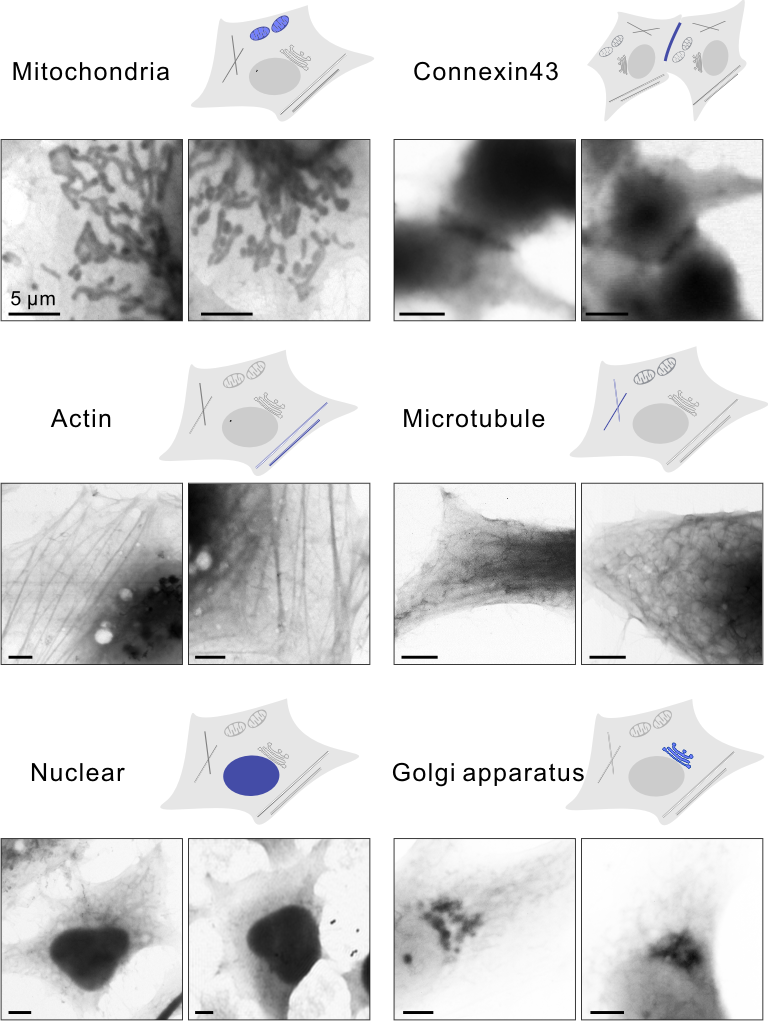


**Supplementary Figure 4. The generality of genetically-encoded X-ray probes for various biotargets.** Each cell was transfected with corresponding plasmids, dyed by DAB, and imaged with X-ray microscopy (They are parallel samples of Fig. 1d). Magnification of the boxed regions were shown on the right of each column. Scale bars: 5 μm.


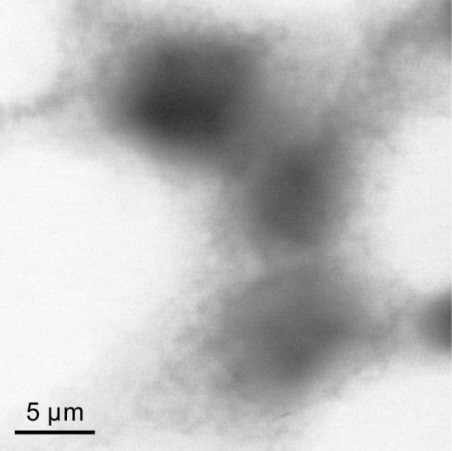


**Supplementary Figure 5. STXM image of untransfected Hek293T cells.** Scale bar: 5 μm.


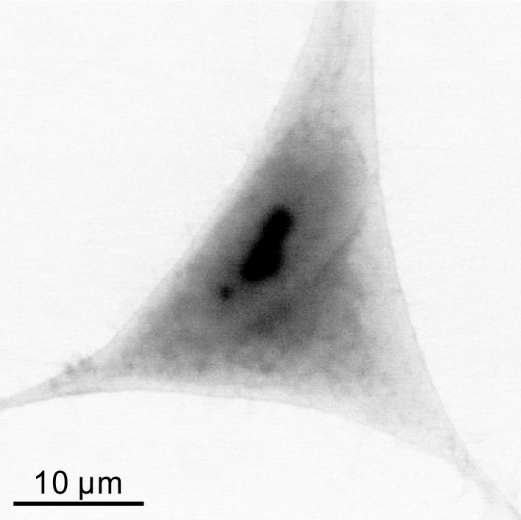


**Supplementary Figure 6. STXM image of untransfected HeLa cells.** Scale bar: 10 μm.


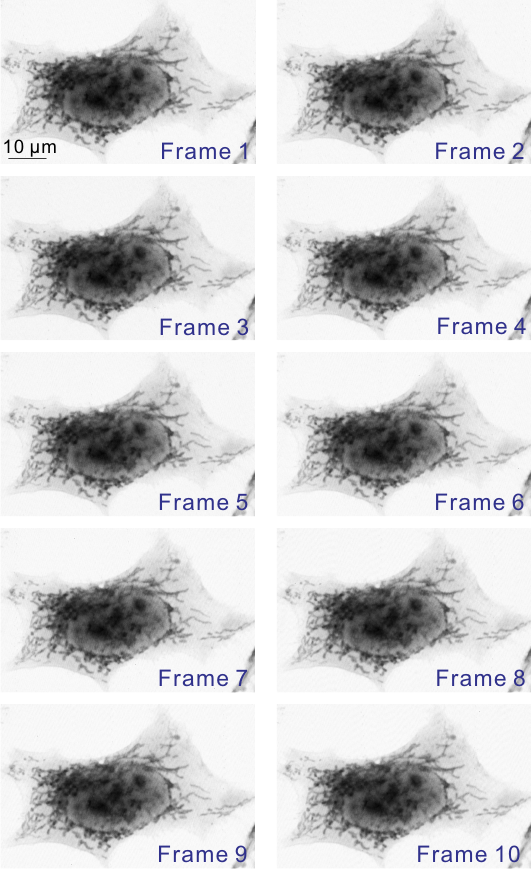


**Supplementary Figure 7. Photostability characterization of genetically encoded tag for protein localization with XRM.** Continuous-10-frame STXM scans of mitochondria in HeLa cells. Scale bar: 10 μm.


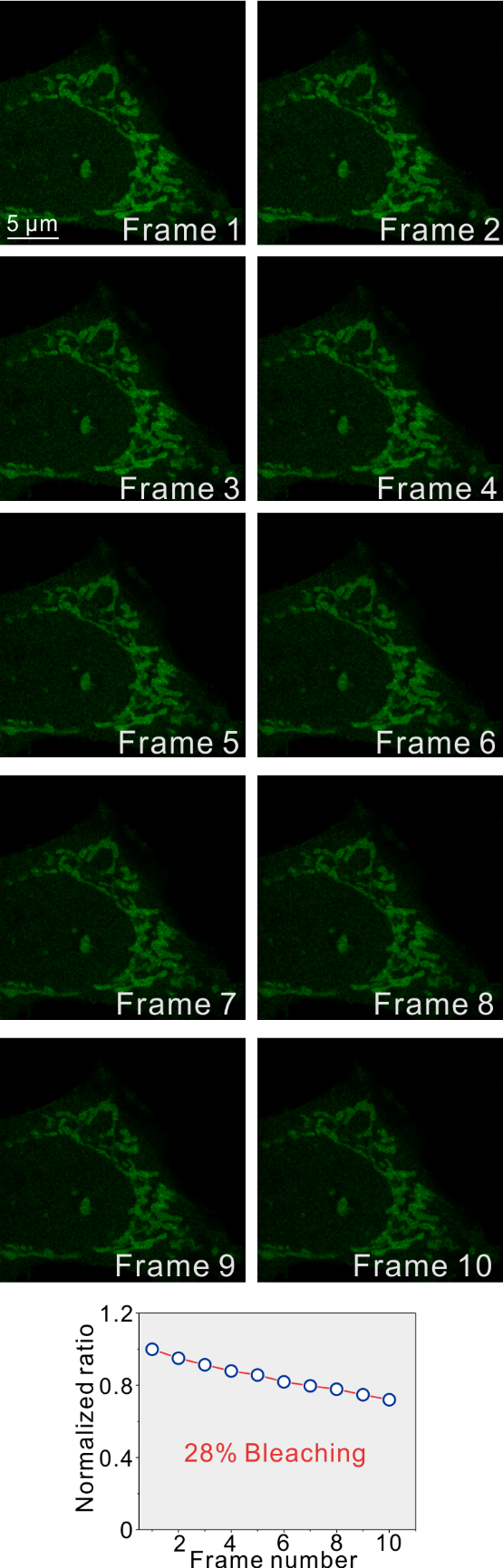


**Supplementary Figure 8. Photostability characterization of endogenous tags for protein localization with fluorescence microscopy.** Continuous-10-frame fluorescence scans of mitochondria in HeLa cells. Scale bars: 5 μm.


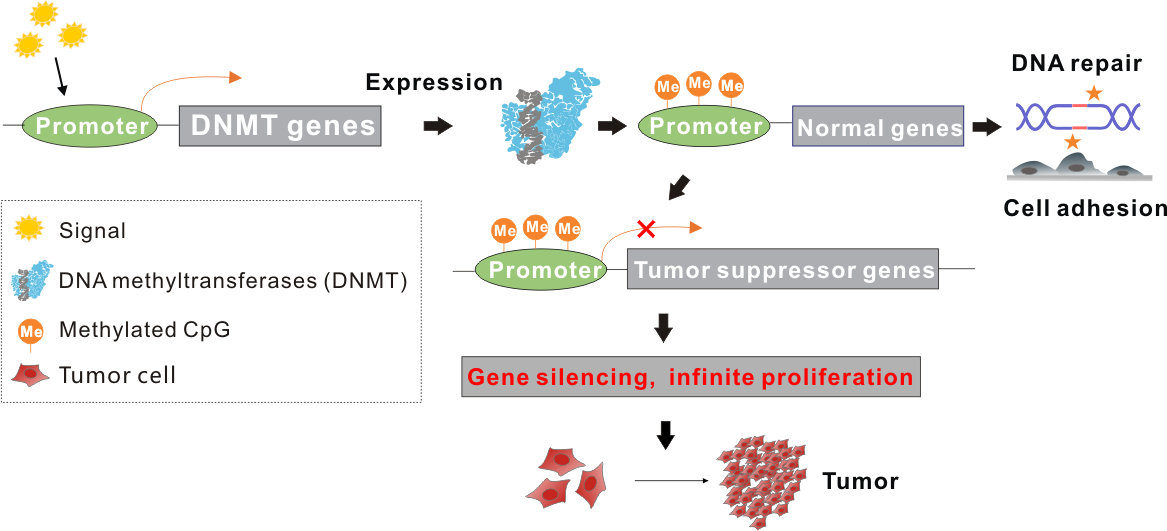


**Supplementary Figure 9. Schematic showing of DNA methylation pathways.**


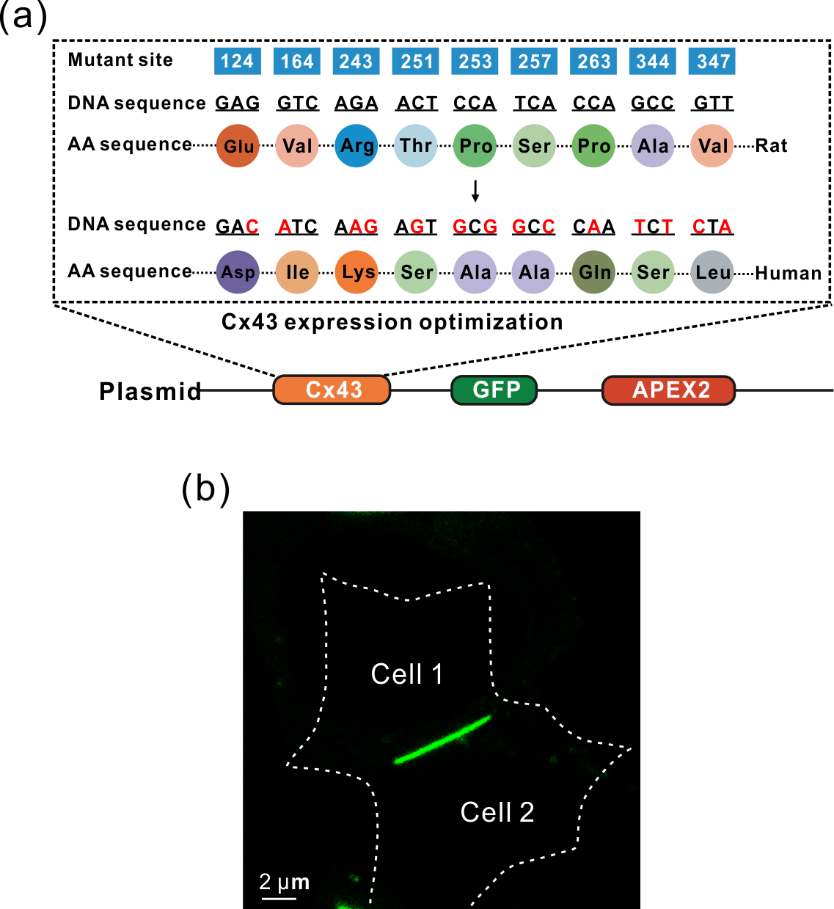


**Supplementary Figure 10. Cx43 expression optimization in human cells. a**, Schematic showing of AA sequence optimization in Cx43-GFP-APEX2 fusion plasmid. **b**, Hek293T cells were transfected with connexin43-GFP-APEX2 plasmids. Confocal images of Cx43 in Hek293T cells. Scale bars: 5 μm.


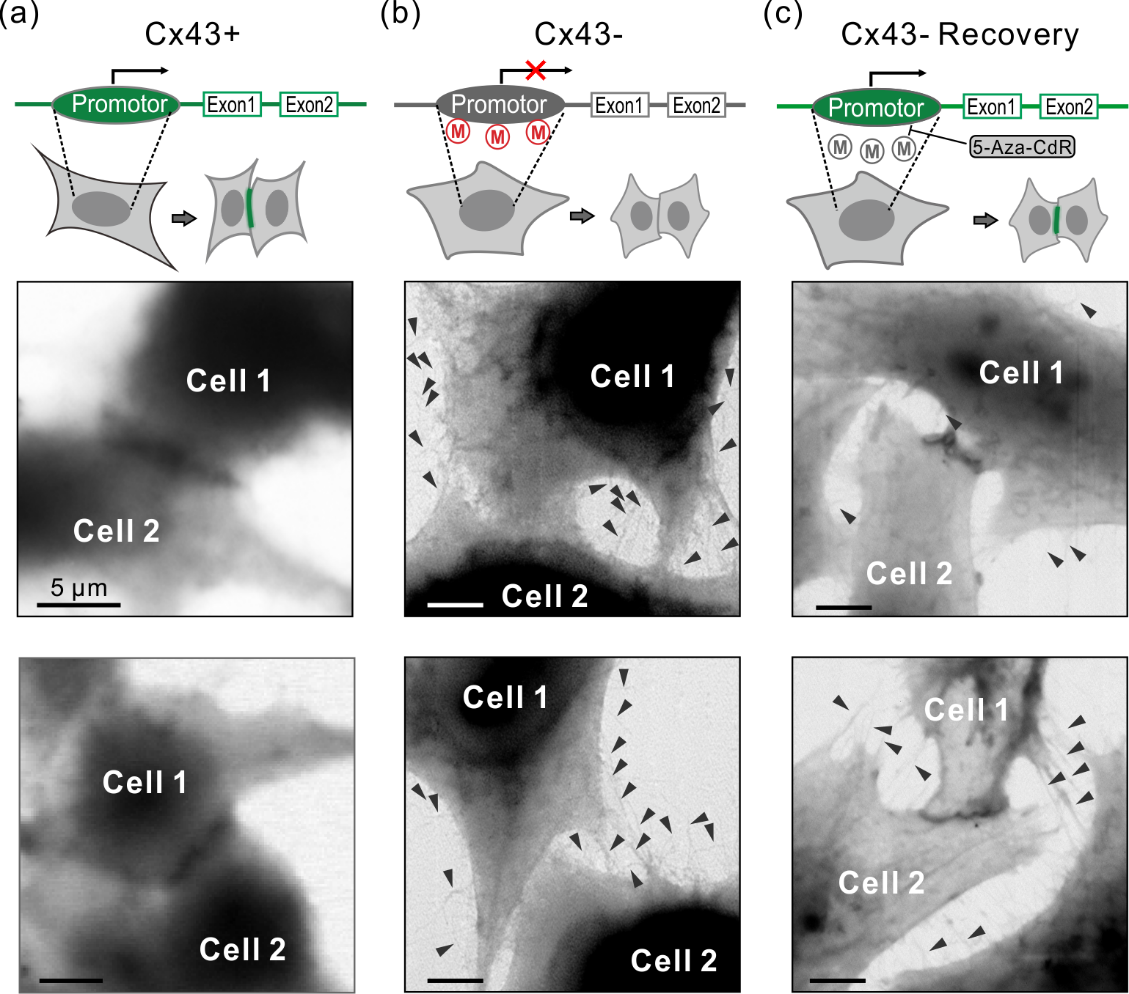


**Supplementary Figure 11. Tracking of regulation of Cx43 expression by DNA methylation in cells with genetically-encoded probes.** For Hek293T cells (Cx43+), they were transfected with hCx43-GFP-APEX2 plasmids; for HeLa cells (Cx43- or Cx43- recovery), they were treated without or with methylation inhibitor 5-Aza-CdR, and then transfected with hCx43-GFP-APEX2 plasmids and dyed by DAB. a-c: a, Cx43+; b, 43-; c, Cx43- Recovery. Upper: Schematic showing of regulation of Cx43 expression by DNA methylation in cells. Lower: X-ray images of Cx43 in cells. (They are parallel samples of Fig. 3a-3c). White arrows indicate the primary cilia on the surface of HeLa cells. Scale bars: 5 μm.


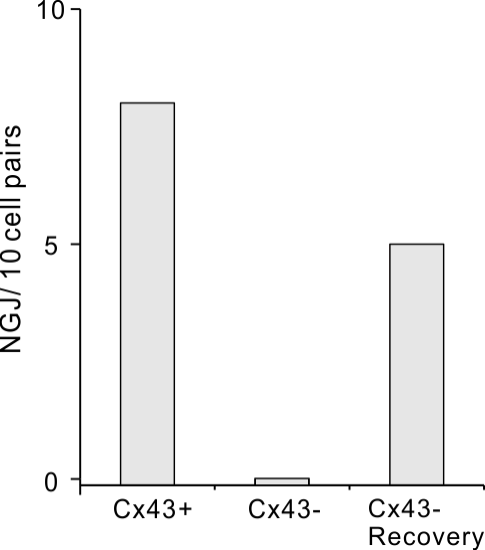


**Supplementary Figure 12. The number of gap junction-positive cell pairs (NGJ) in treated cells.** For Hek293T cells (Cx43+), they were transfected with Cx43-GFP-APEX2 plasmids; for HeLa cells (Cx43- or Cx43- Recovery), they were treated without or with methylation inhibitor 5-Aza-CdR, and then transfected with Cx43-GFP-APEX2 plasmids and dyed by DAB (10 pairs of cells were randomly selected from each group).


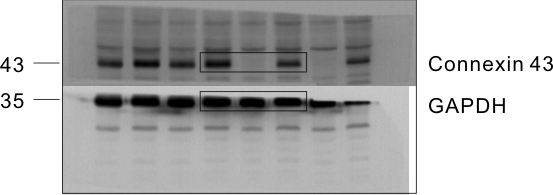


**Supplementary Figure 13. Uncropped scans of western blots in Fig. 3e.**


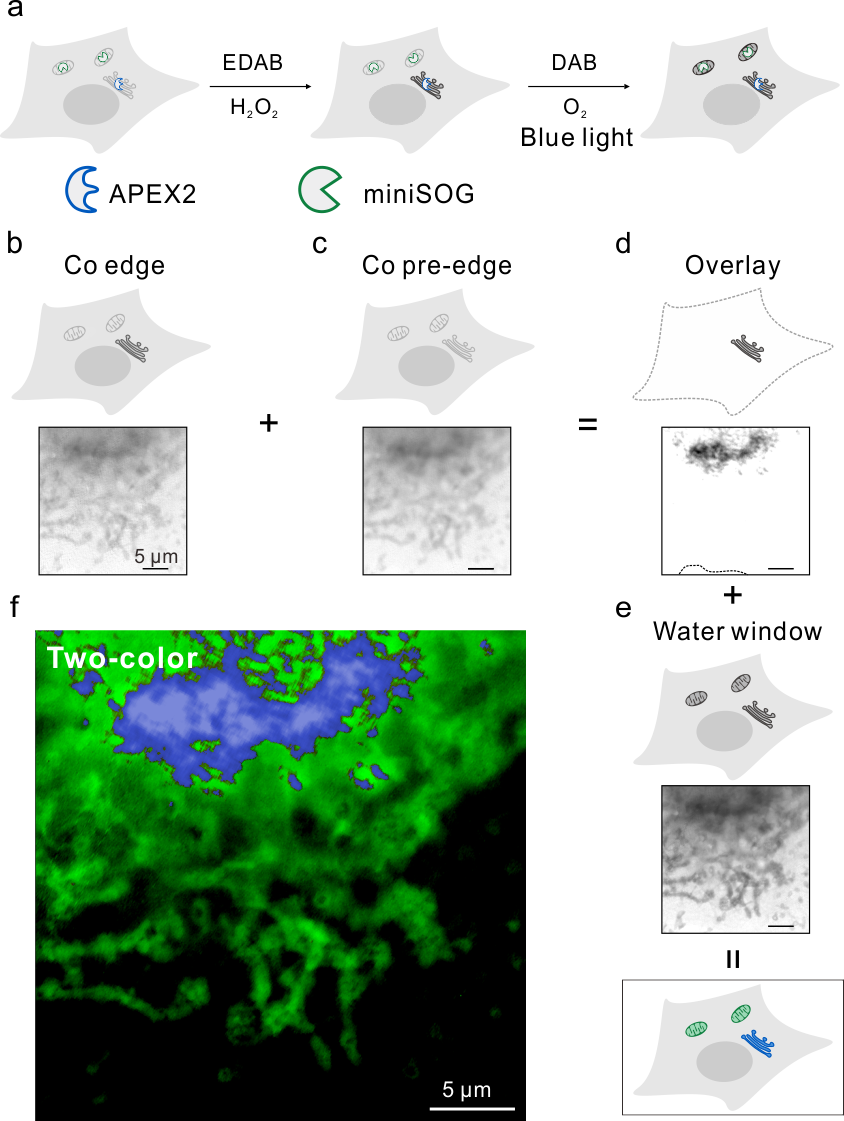


**Supplementary Figure 14. Multi-color X-ray microscopy for simultaneous visualization of two bio-targets.** HeLa cells were co-transfection with Golgi-APEX2 and mito-miniSOG plasmids, and stained with EDAB and DAB in turn. Schematic showing (Upper) and experimental (Lower) of multi-color X-ray microscopy for simultaneous visualization of Golgi and mitochondria. Scale bars: 5 μm. Pseudocolored images of the two-hue representative bio-targets with nanoscale resolution were generated using Image J. Scale bar: 5 μm.


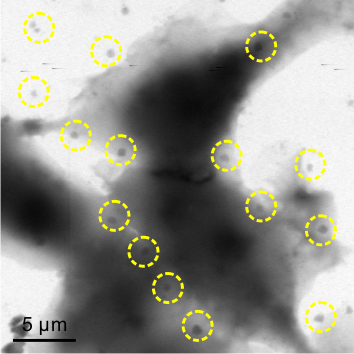


**Supplementary Figure 15. STXM images of Cx43.** Hek293T cells were transfected with hCx43-GFP-APEX2 plasmids. After DAB staining, they were treated with OsO_4._ A number of small black dots, which are impurities introduced by OsO_4_ staining, are indicated by yellow circles. Scale bar: 5 μm.

**REFERENCE**:

1. Lam, SS, Martell, JD, Kamer, KJ*, et al.* Directed evolution of APEX2 for electron microscopy and proximity labeling. *Nat Methods* 2015; **12**: 51-4.
